# Hyperreflective Retinal Foci (HRF): Definition and Role of an Invaluable OCT Sign

**DOI:** 10.3390/jcm14093021

**Published:** 2025-04-27

**Authors:** Luisa Frizziero, Giulia Midena, Luca Danieli, Tommaso Torresin, Antonio Perfetto, Raffaele Parrozzani, Elisabetta Pilotto, Edoardo Midena

**Affiliations:** 1 Department of Ophthalmology, University of Padova, 35100 Padova, Italy; lfrizziero@gmail.com (L.F.); ld.lucadanieli@gmail.com (L.D.); tommaso.torresin@gmail.com (T.T.); antonio.perfetto@studenti.unipd.it (A.P.); raffaele.parrozani@unipd.it (R.P.); edoardo.midena@unipd.it (E.M.); 2 IRCCS—Fondazione Bietti, 00198 Rome, Italy; giulia.midena@fondazionebietti.it

**Keywords:** hyperreflective retinal foci, macular diseases, macular edema, macular degeneration, optical coherence tomography, biomarker, imaging, artificial intelligence, retina

## Abstract

**Background**: Hyperreflective retinal foci (HRF) are small, discrete, hyperreflective elements observed in the retina using optical coherence tomography (OCT). They appear in many retinal diseases and have been linked to disease progression, treatment response, and prognosis. However, their definition and clinical use vary widely, not just between different diseases, but also within a single disorder. **Methods**: This perspective is based on a review of peer-reviewed studies examining HRF across different retinal diseases. The studies included analyzed HRF morphology, distribution, and clinical relevance using OCT. Particular attention was given to histopathological correlations, disease-specific patterns, and advancements in automated quantification methods. **Results:** HRF distribution and features vary with disease type and even within the same disease. A variety of descriptions have been proposed with different characteristics in terms of dimensions, reflectivity, location, and association with back shadowing. Automated OCT analysis has enhanced HRF detection, enabling quantitative analysis that may expand their use in clinical practice. However, differences in software and methods can lead to inconsistent results between studies. HRF have been linked to microglial cells and may be defined as neuro-inflammatory cells (Inflammatory, I-HRF), migrating retinal pigment epithelium cells (Pigmentary, P-HRF), blood vessels (Vascular, V-HRF), and deposits of proteinaceous or lipid elements leaking from vessels (Exudative, E-HRF). **Conclusions**: HRF are emerging as valuable imaging biomarkers in retinal diseases. Four main types have been identified, with different morphological features, pathophysiological origin, and, therefore, different implications in the management of retinal diseases. Advances in imaging and computational analysis are promising for their incorporation into personalized treatment strategies.

## 1. Introduction

The use of medical signs has a long history in medical practice, as objective indicators of a patient’s medical condition observed externally. More recently the use of objective, quantifiable medical signs, named biomarkers, has become of greater relevance in clinical practice and research [1]. Retinal diseases such as age-related macular degeneration (AMD) and diabetic retinopathy (DR) are major causes of vision impairment and blindness worldwide. Despite advances in treatment, late diagnosis and frequent treatment failure remain significant challenges, in both developed and developing countries [2]. Advancements in retinal diagnostic imaging have allowed the identification of medical signs proposed as “biomarkers” of disease development, activity, and response to treatment [3]. Among these, hyperreflective retinal foci (HRF) have emerged as a valuable finding in optical coherence tomography (OCT) imaging, drawing significant attention for their potential role as biomarkers in various retinal diseases. These small, punctate, hyperreflective structures, visible across different retinal layers, have been linked to a wide range of pathological processes, including neuroinflammation, lipid deposition, and cellular debris accumulation. Despite their frequent observation and application in clinical practice, a comprehensive consensus regarding their definition, pathogenesis, and clinical significance is still evolving. Therefore, the aim of this perspective was to review the literature in the field to identify specific types of HRF that may be more clearly associated with distinct pathogenic mechanisms and clinical meaning. This may improve the clinical management of major retinal diseases, by aiding in the identification of disease phenotypes and enabling more targeted therapeutic approaches.

## 2. Materials and Methods

To identify potentially relevant articles in the medical literature, we searched the PubMed database for English-language articles published from January 1980 to 31 December 2024, focusing on studies analyzing HRF morphology, distribution, and clinical correlations using Spectral Domain (SD)-OCT. To ensure comprehensive coverage of the field and account for variability in terminology—for example, “hyperreflective foci” versus “hyperreflective spots”—a broad search strategy was employed. Thus, Pubmed database was queried using the following search terms, both individually and in combination for advanced research: hyperreflective spots, hyperreflective foci, and hyperreflective dots in combination with the terms retina, macula, macular edema, macular degeneration, maculopathy, optical coherence tomography, vein occlusion, diabetes, and artificial intelligence. Additional relevant articles were identified by reviewing the references of selected publications. To identify potentially relevant articles to be included in this perspective, two investigators reviewed each paper. We included the most significant, commonly referenced, and highly regarded publications. All articles cited in the reference list were thoroughly examined by the authors.

## 3. Results

Using the main search terms (hyperreflective spots, hyperreflective foci, and hyperreflective dots), a total of 1004 articles were initially identified. Refining the search with secondary terms (e.g., retina, macula) narrowed the selection to 674 articles. The terms optical coherence tomography and artificial intelligence were also used separately in combination with the main terms to identify any additional relevant articles, resulting in a total of 695 articles. After removing duplicates, non-English publications, and articles clearly unrelated to the main topic—based on title screening—a total of 385 articles were reviewed in full. Priority was given to articles addressing the pathogenesis, behavior, and morphological characteristics of HRF. Cohort studies, case–control studies, and systematic reviews were preferred over case reports or small case series. In the final selection, 83 articles were included in this paper. Additional references were drawn from the bibliographies of the selected articles and from further searches conducted during the writing process.

### 3.1. Biomarkers

#### 3.1.1. Definition of Biomarker

One of the first definitions of biomarker was provided by the World Health Organization (WHO) in its report on the validity of biomarkers in environment risk assessment, stating: “almost any measurement reflecting an interaction between a biological system and a potential hazard, which may be chemical, physical, or biological. The measured response may be functional and physiological, biochemical at the cellular level, or a molecular interaction.” [4]. Similarly the National Institutes of Health Biomarkers Definitions Working Group defined a biomarker as “a characteristic that is objectively measured and evaluated as an indicator of normal biological processes, pathogenic processes, or pharmacologic responses to a therapeutic intervention.” [5]. This definition distinguishes biomarkers from medical symptoms, which rely solely on the patients’ perception of health or illness. Conversely, the term parameter should be used when a sign has not been adequately quantified. Both biomarker definitions emphasize the role of biomarkers as tools to detect and evaluate a clinical process but also to monitor its changes, whether due to treatment or not. Therefore, biomarkers may help identify normal or pathological biological processes, support the follow-up of that process, also in a quantitative way, and serve as valuable tools in clinical practice. Moreover, they can function as surrogate endpoints in clinical research [1,4,5].

#### 3.1.2. The Use of Biomarkers in Retinal Disorders

The improvement of our knowledge in imaging chorioretinal biomarkers has become a major focus of research in recent years. A multimodal approach in ocular and systemic diseases involving the eye requires the integration of different clinical biomarkers, which must be well characterized to correctly predict relevant clinical outcomes across a variety of treatments and populations [1,6]. Currently, the most widely used imaging test in ophthalmic practice is OCT. This noninvasive imaging technique provides cross-sectional images of the choroid and retina, close to an in vivo “optical biopsy”, and it allows the identification of morphologic features related to normal and pathological processes, some of which are being studied as possible appropriate biomarkers [7].

#### 3.1.3. Nomenclature

In recent years, one of the most discussed OCT-based morphological features, proposed as a retinal biomarker, is the presence and the amount of some retinal hyperreflective elements, called HRF for the first time by Khanifar et al. [8]. These authors suggested that these hyperreflective foci overlying drusen represent migrating RPE cells [8,9]. Coscas et al. reported the presence of hyperreflective foci, which they originally called hyperreflective dots (HRDs), scattered throughout all retinal layers, primarily around fluid accumulation in the retinal cystoid spaces, in exudative age-related macular degeneration (AMD) and interpreted them as signs of inflammatory cells activation [7]. These elements have since been identified in other different disorders such as diabetic retinopathy (DR), where hyperreflective retinal foci (sometimes defined as spots or dots), defined as small, punctiform, white lesions, were also detected in its early stages through OCT [10]. Bolz et al. also described hyperreflective foci in diabetic macular edema (DME), correlating them to subclinical lipoprotein extravasation following breakdown of the inner blood–retina barrier [11]. Gelman et al. proposed that hyperreflective material on OCT, especially when observed in a “pearl necklace” configuration, may be more consistent with cells, namely lipid-laden macrophages, along the walls of the cysts [12].

The detection of these hyperreflective elements in different retinal disorders and their heterogeneity in distribution and morphology even in the same disease gave rise to several denominations creating confusion as well as difficulties in literature searches [13]. The term “spot” refers to a small area visibly different (as in color, finish, or material) from the surrounding area. It frequently refers, in the digital era, to the image created from a light beam pointed onto a screen. The term “focus” (from latin, literally “fire, fireside”) is considered a point at which rays (of any kind—light, sound, or even in a figurative sense) meet or from which they appear to come and spread. In medicine, it is often used to describe the source of an infection. Finally, the term “dot”, which was first used referring to retinal hyperreflective elements, preferentially indicates a small spot or mark consisting of a single point. It is used in writing, mathematics, music, or Morse code, i.e., dot com, dot per inch (DPI), etc. [14,15]. Therefore, the terms “foci” and “spots” appear to well fit the description of these small, hyperreflective, round, discrete elements which stand out in OCT scans and are increasingly considered new biomarkers of retinal disorders and predictors of response to treatments. In the recent literature, the term “foci” has been more frequently and continuously used; therefore, we will use this denomination to encompass previous definitions of dots and spots.

To review and describe the clinical features and the interpretation of HRF in different retinal disorders, we reviewed the literature and reported the most relevant clinical characteristics of HRF in different retinal disorders, according to their pathophysiological interpretations.

### 3.2. Vessels and Vascular Abnormalities

The high resolution of current OCT devices has increased the possibility of visualizing tiny intra-retinal structures, and some hyperreflective elements on OCT B-scan may correspond to retinal structures of vascular origin, namely blood vessels and their possible abnormalities, such as dilations, telangiectasias, and microaneurysms [13,16,17,18]. This is supported by their typical localization within the retinal nerve fiber layer (RNFL), ganglion cell layers, and the inner nuclear layer, where the superficial and deep capillary plexuses are located, respectively [19]. It has been shown that these types of OCT features tend to have specific characteristics, which can help distinguish them from HRF of other origin. In particular, their size is usually greater than 30 μm [13]. Moreover, the reflectivity is intermediate, similar to the RNFL, although some variability has been reported, in particular when considering microaneurysms [18]. Finally, they are usually associated with the presence of back shadowing. One of the first imaging techniques that enabled the better definition of these vascular structures has been the en face modality of OCT. This multi-sliced technique, in fact, allows a precise localization (along the C- axis) of intra-retinal elements visualized on B-scan. Therefore, HRF may be precisely localized in the retinal layers and correlated to fundus findings, such as vessels, microaneurysms, and hard exudates [17]. More recently, the advent of OCT angiography has further confirmed the correlation between specific hyperreflective elements on structural OCT scans and the presence of a vessel or its possible abnormality [13,18,20]. However, HRF modification after cataract surgery (see below) did not correspond with capillaries’ quantitative modifications at different follow-up times, as documented using OCT angiography, suggesting the presence of other pathophysiological explanations for HRF changes [20]. Therefore, when analyzing hyperreflective intraretinal findings, HRF with the above-mentioned characteristics should not be considered as a biomarker of a pathophysiological phenomenon, but rather as the typical OCT appearance of vascular structures.

### 3.3. Retinal Neuroinflammation

Since the earliest observations of HRF, it has been hypothesized that they may represent aggregates of microglial cells, as supported by histological post mortem studies of diabetic and healthy human retinas where microglia cell findings seem to mimic the location of HRF on OCT [7,21,22,23,24,25,26]. In the healthy retina, microglia cells are primarily located in the inner retinal layers, in a ramified resting status [24]. When activated, for example, in diabetes mellitus, they turn into an “ameboid” form, gain motility, and migrate towards the outer retina, and they release an increased amount of inflammatory and vasoactive molecules, such as cytokines, chemokines, and growth factors, initiating a local inflammatory cascade [24,27]. HRF have been detected in healthy and diabetic eyes without clinical DR, mainly located in the inner retina [10,28]. Vujosevic et al., in fact, demonstrated the increased presence of HRF in diabetic patients compared to non-diabetic subjects, even without detectable retinopathy, suggesting their early appearance in the development of diabetic retinal damage [10]. Moreover, with the progression of retinopathy, and especially when diabetic macular edema (DME) occurs, HRF appear all over the retinal layers, mimicking the migration of microglia cells towards the outer retina [10,27,29,30].

In other OCT studies on macular edema of different origins, also associated with foveal serous retinal detachment (SRD), HRF have been demonstrated in proximity to the external limiting membrane (ELM) [31,32]. These findings seem to suggest that the location of HRF on OCT reflects microglia cell positions in vivo and that aggregates of microglia cells may be simply detected with non-invasive OCT scans [29]. It has also been reported that the number of HRF increases in the phenotype of DME associated with foveal SRD, compared to DME without SRD [28]. Proteomic studies on aqueous samples from eyes with DME and SRD confirmed an increased concentration of inflammatory mediators, especially interleukin 6, thus reinforcing the presence of a predominant inflammatory pathogenesis in this disease phenotype [33].

The presence of HRF has also been noted in exudative AMD, and small HRF were supposed to be activated microglia involved in an inflammatory response [7]. These were differentiated from another type of HRF, also present in AMD, corresponding to RPE cells, with different location, characteristics, and behavior compared to small HRF corresponding to microglia cells [34,35]. HRF were also described in geographic atrophy (GA), particularly in eyes with unilateral GA (and macular neovascularization in the fellow eye) compared to bilateral GA, after exclusion of larger HRF with other OCT features probably corresponding, for example, to pigmented cells migrating into the retina [36]. In these eyes, HRF corresponding to microglia appear as a key factor in characterizing these two different GA phenotypes [36].

Turgut and Yildirim analyzed the presence of HRF in several patients affected by different maculopathies, excluding those with DME or retinal vein occlusions [31]. Interestingly, these authors willingly excluded maculopathies with the presence of hard exudates in order to avoid bias; still they found the presence of HRF, once again confirming that inflammatory cells may be responsible for these intra-retinal findings on OCT [31].

More recently, Mat Nor et al. correlated OCT examination with histological immunolabelling in an animal model, showing that small HRF (called dots in this paper) seen in OCT images of the retina are associated with a local microglial response [37]. In another study, the examination of the same eyes using both OCT and angiography showed that some HRF presented decorrelation signals, hypothesized as an expression of morphological changes in microglia/macrophages or intracellular organelles containing highly reflective material [38,39].

Vujosevic et al. showed that some specific OCT characteristics of HRF, which may reasonably represent microglia cells, may be identified and include the following: a maximum diameter of less than 30 µm, an intermediate reflectivity, similar to RNFL, and the absence of posterior back shadowing. Their location may be anywhere in the retinal layers, but the foci within the wall of cystic spaces are unlikely to represent microglia cells [13] (Table 1).

HRF have shown to reduce in number after treatments with both anti-vascular endothelial growth factor (VEGF) and steroids in different types of maculopathy [32,40,41,42,43]. After treatment, a reduction in retinal inflammation is expected, and, in the retinal environment, this is represented by a de-activation of microglia cells, suggesting that the number of HRF is a reliable biomarker of retinal response to treatment. It is also noticeable that in other types of inflammatory maculopathies, such as radiation maculopathy and post-surgical macular edema, an increased number of HRF has been found [20,44,45]. In particular, in eyes treated with radiotherapy, an increase in HRF number has been correlated with the subsequent development of macular edema and the severity of the edema, suggesting their role as a predictive biomarker of maculopathy development and disease severity [45,46]. Increased HRF have also been found in patients affected by relapse-onset multiple sclerosis at baseline, and a strong association between HRF and cortical pathology in relapsing–remitting multiple sclerosis seems to suggest an underlying common immunopathologic mechanism [47,48,49]. In patients affected by non-inflammatory neurological disorders, HRF correlated with intrathecally produced monocyte/microglia-derived cytokines, confirming their inflammatory nature [50]. Pilotto et al. also described an increased number of HRF after uncomplicated cataract surgery, a purely inflammatory condition [20].

More recently, the appearance of hyperreflective foci in the choroid has been reported in diabetic eyes, correlating with the severity of DR and the presence of DME [51,52]. They have been hypothesized to represent aggregates of inflammatory cells within the choroid or migrating from the retina, particularly when the ELM and ellipsoid zone are disrupted, as visualized on OCT [51,52].

### 3.4. Exudation

In 2009, Bolz et al. hypothesized that HRF in patients with diabetic macular edema (DME) could represent an early manifestation of lipid extravasation [11]. These HRF were distributed throughout retinal layers, including the subretinal space. When looking carefully at the HRF considered by Bolz et al., many of them were located within the walls of intra-retinal cysts. Other HRF were highly reflective or clustered in a confluent pattern of elevated dimension [11]. Hyper-reflective structures detected on OCT scans in other retinal disorders have also been attributed to exudative phenomena. For example, Gelman et al. reported a new OCT sign, named by the author as “necklace sign” [12]. This sign was described as the presence of hard exudates in a contiguous ring around the inner wall of cystoid spaces in the outer plexiform layer (OPL), detected in different types of chronic exudative maculopathies such as AMD, DME, retinal vein occlusion, retinal arterial macroaneurysm, and Coats disease. These authors speculated that the hyperreflective material consisted of lipoproteins or lipid-laden macrophages [12]. Other researchers further suggested that HRF may represent a morphologic sign of the accumulation of intra-retinal fluid and lipid extravasation in cases of macular edema secondary to diabetes or retinal vein occlusion and in subjects with macular degeneration, even if HRF detected on OCT are not clinically visible on fundus examination as hard exudates [53,54,55,56,57]. These foci were reported to be of variable dimensions and, even when their specific features were not specifically defined, looking at the reported imaging, they appeared highly reflective, with relatively high dimensions (>30 µm) and an inconsistently homogenous distribution [53,54,55,56,57,58]. Ogino et al. speculated that the scattered HRF in the affected area of branch retinal vein occlusions represented leakage of blood constituents, whereas the confluent spots in the unaffected areas were associated with absorption of water and solutes [56]. They hypothesized that lipid-laden cells, specifically macrophages, tend to accumulate around the OPL [56,59]. The OPL was supposed to have a higher affinity for extravasated lipids and proteins, thus attracting macrophages. Ogino et al. concluded that further clinico-pathologic investigations with OCT would clarify the relationship between the formation of hard exudates, macrophages, and HRF [56]. Also, Pang et al., in AMD, found an association of the “onion sign” (layered, hyperreflective lines located below the retinal pigment epithelium) with the presence of HRF. Although HRF were associated with exudative phenomena, the histologic examination of two donors in this study revealed that retinal hyperreflective foci correlated with different types of cell populations, including microglia cells, migrating from inner to outer retina with macrophagic functions, and anteriorly migrating RPE cells [59].

### 3.5. Degeneration

As previously mentioned, HRF were firstly reported as a OCT morphologic entity in AMD [7,8]. Coscas et al., among other hypotheses, excluded the pigmentary nature of some HRF since they did not find any correspondence to HRF in blue-light fundus photography and autofluorescence [7]. Subsequently, HRF were further described in AMD, and their presence was linked to other pathophysiologic hypotheses, such as a blood–retinal barrier dysfunction, and associated with a poor visual prognosis [60]. More recently, the comparison between OCT B-scan and histologic sections from ex vivo eyes affected by drusenoid pigment epithelium detachment (PED) in AMD showed that simple and complex groups of HRF may correspond to small and large clusters of RPE cells, respectively [61]. Specifically, Balaratnasingam et al. correlated HRF with nonuniform, sloughed, single cells or swarms of fully pigmented RPE cells, located in the outer nuclear layer, Henle fiber layer, or forming a ring around an inner retinal capillary and also migrating anteriorly to the ganglion cell layer [61]. Moreover, PED associated with HRF was shown to have a greater tendency to progress to advanced AMD, suggesting a role of these HRF in AMD progression, particularly when showing an inward movement over time [61,62]. In AMD, RPE cells are thought to have the ability to transdifferentiate into phenotypes with new potentials, including motility and expression of inflammatory markers [62,63]. The migration of RPE cells from their original location has been related to the development of both choroidal neovascularization and GA, two different pathways deriving from the same pathological original spectrum. [38,62].

The RPE origin of HRF has also been confirmed in acquired vitelliform lesions where HRF were located above the lesion, in proximity to the fovea, and migrated anteriorly and along the Henle fiber layer. Moreover, HRF migration seems to be preceded by the disruption of the ELM and ellipsoid zone, suggesting the creation of a conduit for the movement of RPE away from the subretinal space [63,64,65]. More recently, a correlation study between OCT and histopathologic images in type 3 neovascularization showed the presence of different types of HRF [34]. HRF were identified in OCT B-Scan at different distances, superior to the center of the type 3 neovascularization, and have been proven to correspond to two different types of cells, as hypothesized also in other AMD studies [34,35,38,59]. The first type corresponds to nucleated RPE cells, which are located in the OPL and inner nuclear layer (INL), superior to neovascularization, and, as previously mentioned, they can be found next to a capillary. These intra-retinal RPE cells cluster mostly within 150 µm of the neovascularization and could correlate to hyperautofluorescent areas. Different intra-retinal lipid-filled cells were also located near the neovascularization, superior to it, but farther than RPE cells (270–1350 µm), still in the exudation area. This second type of cells seems to correspond to microglia cells, activated secondary to retinal damage in degenerative retinal disorders, as well as in vascular disorders, as mentioned above [34,35].

Therefore, although the full characterization of HRF in AMD is still evolving, those corresponding to RPE cells are usually located in the outer retina, may migrate anteriorly, and exhibit high reflectivity (similar to RPE), back shadowing, and variable dimensions and morphology [35,61,66].

### 3.6. Artificial Intelligence Application

In recent years, artificial intelligence in medicine, and notably in ophthalmology, has assumed growing relevance [67]. The automatic detection of pathology-related features seems to be the most promising approach in disease treatment and monitoring. In this context, HRF appear to be a suitable entity to be analyzed with an artificial intelligence approach [67,68]. In fact, in analyzing HRF as a potential biomarker in different diseases, and even in the same disease, the existing body of literature has been inconsistent sometimes [69]. This may be due to variations in the imaging device used, image quality, and manual segmentation of HRF. Therefore, artificial intelligence techniques for quantifying HRF appear as a way to overcome at least some limitations of previous studies, particularly the standardization of analysis [70]. Algorithms have been developed using various techniques for automated evaluation of HRF, using variable dataset numerosity, different amounts and types of labeling, and different layer segmentations [68,70,71,72,73,74,75,76,77]. HRF automatic detection has been proposed as a tool to help identify healthy and pathological scans in DR, alongside other biomarkers such as neuroretinal detachment, even if using a limited set of labeled images for model training [74]. Other algorithms have shown good performances in identifying the area and amount of hyperreflective intraretinal elements, with some variability among studies, probably also due to the different elements analyzed, which may have different degrees of reflectivity, dimensions, localizations, and characteristics, as above mentioned, particularly when analyzed in different diseases [68,72,73,77,78,79,80]. Bogunovic et al. proposed a predictive model for AMD progression including HRF segmentation. They noted how the use of different types of HRF pooled together may underestimate their role in the prediction of disease progression [79]. Some algorithms have incorporated strategies to exclude, for example, retinal vessels, combining infrared images with OCT scans or using the presence of back shadowing and location [76,81]. Wang et al. recently proposed a deep learning-based approach to detect and quantify HRF in OCT images of DME patients [70]. They included a wide range of HRF dimensions, concluding that different subtypes exists with different characteristics and behavior, confirming previous clinical studies [70]. Huang et al. proposed an algorithm to detect and quantify HRF, distinguishing two types of them, namely hard exudates and small foci (hypothesized to be activated microglia) [82]. Recently, algorithms able to identify and quantify HRF with other major retinal biomarkers have been proposed [72,83]. Midena et al. reported an algorithm specifically developed and validated for DME, able to detect and quantify a specific type of HRF clinically associated with microglia cells, intraretinal and subretinal fluid, and the integrity of the ellipsoid zone and external limiting membrane [72].

## 4. Discussion and Final Proposal

In the last decade, the technological advancement of OCT devices has significantly improved the detection of even small HRF in the macular region. Their presence has drawn increasing attention from retina specialists as a potential imaging biomarker across a range of macular disorders. The correlation of HRF with disease severity, both from a morphological and functional point of view, has been extensively reported, as well as their possible predictive role for disease development and response to treatment in different macular diseases, with variable results [38]. For example, in DME, HRF, especially in the outer retinal layer, have been linked with both photoreceptor damage and visual impairment in eyes treated with anti-VEGF [41,64,84,85,86]. Moreover, a higher number of HRF was correlated to the risk of early recurrence after treatment with a intravitreal dexamethasone implant [87]. In macular edema secondary to retinal vein occlusion treated with intravitreal dexamethasone or ranibizumab, HRF have been associated with a poorer visual outcome, but a better response to the dexamethasone implant [43,88]. In intermediate AMD, increased HRF volume has been associated with reduced retinal function [89,90], and HRF have been suggested to have a predictive role for AMD progression [91,92]. Moreover, the quantity and location of HRF in neovascular AMD have shown a prognostic value in intravitreal anti-VEGF treatment [93].

A recent systematic review focused on HRF in DME showed that HRF number decreases with treatment, but the results in terms of treatment response prediction are not uniform among studies, requiring more investigations with more uniform approaches [69]. The presence and number of HRF at baseline have been variably correlated to poor or good visual final acuity [84,85,94,95,96]. This is in accordance with the observation that many types of HRF may be detected on OCT scans, and they cannot be considered a single entity, nor can all HRF can be considered as true biomarkers [13]. For example, Zur et al. and Chatziralli et al. reported that the presence of HRF was inversely correlated with final visual acuity in eyes treated with intravitreal steroids for DME [97,98]. Conversely, Hwang et al. found that a larger number of HRF was associated with a better outcome in eyes treated with dexamethasone [99]. However, HRF with different features were evaluated in these studies, correlating them to lipoprotein extravasation in the first case and to neuroinflammation in the last one [97,98,99].

Since many types of hyperreflective findings may be detected on OCT, a correct classification of HRF is mandatory to correctly speculate on their nature and their clinical implications [13]. The classification and standardization of HRF may offer a pathway toward more consistent interpretation and improved diagnostic accuracy across practices. By establishing clear morphological and locational criteria, clinicians can better differentiate between physiological and pathological HRF, leading to earlier detection of disease activity, more tailored treatment decisions, and improved monitoring of disease progression. Standardization also facilitates integration with artificial intelligence tools, enabling automated analysis and broader screening potential, particularly in underserved regions.

On OCT scans, the retinal vessels or other vascular abnormalities may be responsible for the appearance of HRF, which may be defined “Vascular” HRF (V-HRF). These HRF represent the image of a vessel section and, obviously, cannot be considered a biomarker of a macular disease. They are characterized by intermediate reflectivity, a dimension >30 µm, the presence of posterior shadowing, and their location.

Another different type of HRF has been recognized, which does not correspond to any ophthalmoscopic findings and can be visualized in the inner retina but also in the outer retina, namely the ONL, where retinal capillaries are absent, as confirmed using OCT angiography. These HRF seem to have specific features, which suggest their correlation with microglia cells [21,22,23,100]. These are considered the resident immune cellular component of the retina, predominantly localized in the inner retinal layers, in a ramified resting status. In eyes with DR, microglia cells are hypertrophic, increased in number, and scattered through all retinal layers [27,29]. Proof of microglia cell activation in retinal disorders also derives from studies showing an increase in microglia-related inflammatory mediators in ocular fluids [26,101,102,103]. These cells, especially when clustered, may be detected in vivo with high resolution OCT, even in normal eyes, and they are characterized by small dimensions (<30 µm), intermediate reflectivity, the absence of posterior shadowing, and locations anywhere in the retinal layers, according to the pathophysiology of the specific retinal disorder [13,69,104]. We describe these HRF as “neuro-Inflammatory” HRF (I-HRF).

HRF have also been hypothesized to represent hard exudates, lipid-filled macrophages monocytes, or extravasated lipid components. These HRF are distinguishable from previous HRF due to their higher dimension (>30 micron) and reflectivity (similar to RPE), presence of back shadowing, non-uniform distribution, more evident around the outer plexiform layer, location within the cyst wall, and, sometimes, correlation with a typical yellow appearance on fundus examinations [13,69]. We propose to call them “Exudative” HRF (E-HRF). They have been correlated with worse visual prognosis after treatment, particularly when located in the macular center and in clusters [105].

The identification and interpretation of HRF in degenerative macular disorders has gained great interest. In fact, their presence, for example, in AMD, has been correlated with a more rapid progression of the disease, both for neovascular AMD and GA [91,106]. Even in AMD, different types of HRF have been identified and histologically proven. HRF corresponding to migrating RPE have been described as well-circumscribed lesions with high reflectivity similar to RPE, often with back shadowing, located within the neurosensory retina, particularly in the outer nuclear layer, often overlying an area of drusen, with variable conformation [35,107]. We suggest calling them “Pigmentary” HRF (P-HRF).

**Figure 1:**
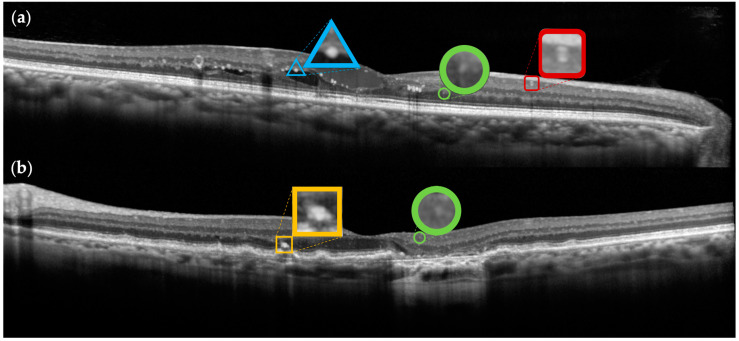
Two optical coherence tomography B-scans, acquired in the high-resolution (HR) modality and >90 ART, of an eye affected by diabetic macular edema (**a**) and an eye affected by age-related macular degeneration (**b**). V-HRF is included within a red square with rounded corners. E-HRF is included within a blue triangle. P-HRF is included within orange square. I-HRF are included within green circles. All HRF have also been magnified for clarity.

In the era of artificial intelligence, imaging biomarkers, such as HRF, have the possibility to become a useful tool in the management of macular diseases, also contributing to therapeutic decision making [108]. In order to develop applicable algorithms, we need to provide uniform and standardized data to the AI systems, i.e., specific characteristic identifying the type of HRF to analyze, as recently underlined by Midena and Frizziero [58,70,82]. At present, developed algorithms are not clearly comparable with each other because they often use different datasets, evaluation metrics, image preprocessing methods, and annotation standards, making direct performance comparisons unreliable and limiting reproducibility across studies. In future studies, standardization of these elements and their clear reporting, with the use of high-resolution images, would improve the clinical utility of the results as support for clinician decision making. To allow the correct interpretation of results, we propose distinguishing HRF as follows: vascular (V-HRF), exudative (E-HRF), pigmentary (P-HRF), and neuroinflammatory (I-HRF) (Figure 1).

The different features of HRF may also require different methods of evaluation, such as the count of single detected elements or an area or volume for bigger HRF with non-uniform dimensions [10,109]. Moreover, image quality across different OCT devices but also using the same OCT device is a significant issue in the standardization of the identification of HRF. The use of high-resolution OCT line B-scan passing through the fovea, with an axial resolution of 3.9 µm, should be recommended in order to detect and fully characterize all HRF types [72]. The need for enhanced OCT images to improve the perception and interpretability of HRF has been reported, and different scan resolutions may strongly discriminate the ability to identify structures as tiny as a micron in size [110]. Therefore, at present, the use of different scan resolutions, scan areas of analysis, methods of quantification, and HRF characteristics make the majority of studies not comparable and the evidence not applicable in clinical practice in a standardized way.

One limitation of this paper is the absence of a metanalysis to systematically compare the results of the studies on HRF. However, this was not the aim of the study, considering that the majority of studies are not comparable with each other because of the lack of uniformity in the approach to HRF, as already reported also by other authors [58,69]. This is also why we chose to only partially address the therapeutic implications of HRF, given that the results in this area are not directly comparable [69]. This observation highlights the need for a more refined categorization of HRF, which is the main topic of this paper.

## 5. Conclusions

Nowadays, it is evident that more than one type of OCT hyperreflective entity exists, differing both in appearance and underlying biological nature. Considering all HRF as a single entity may be misleading. Reviewing the scientific literature on this topic, four major hypotheses on the nature of hyperreflective spots on OCT have been identified: vascular elements, i.e., retinal vessels and vascular abnormalities (V-HRF); cluster of activated microglia cells, as a biomarker of retinal neuroinflammation (I-HRF); extravasated lipids, proteins, or phagocytizing cells, as a sign of vascular compromise (E-HRF); and RPE-derived cells in retina degenerations (P-HRF). Numerous studies have highlighted the clinical relevance of HRF in a range of macular diseases, in terms of diseases severity, progression, and response to treatment. However, to effectively translate this evidence into clinical practice we need to clearly distinguish and analyze each HRF type. Future research should prioritize the validation of HRF classification systems through the development of automated detection algorithms incorporating morphological criteria and longitudinal studies exploring the prognostic value of each HRF type in specific diseases. Investigating the molecular and cellular correlates of each HRF type through multimodal imaging and histopathologic correlation will further strengthen their utility as imaging biomarkers. Refining our understanding and classification of HRF will not only enhance diagnostic accuracy and disease monitoring but also open new pathways for personalized treatment strategies, particularly in the emerging context of AI-driven retinal care.

## Figures and Tables

**Table 1:** HRF characteristics.

Denomination	Origin	Dimension	Reflectivity	Back-Shadowing	Location
Vascular V-HRF	Vascular structures	>30 µm	Intermediate, similar to RNFL	Yes	Inner retina
Exudative E-HRF	Exudation/extravasated lipoproteins and/or proteins	>30 µm	High, similar to RPE	Yes	Non-uniform distribution, particularly pronounced around the outer plexiform layer and also observed along the cyst walls
Pigmentary P-HRF	Degeneration/RPE- derived cells	Variable dimension and conformation	High, similar to RPE	Yes	Predominantly in the outer retinal layers, especially overlying drusen
Inflammatory I-HRF	Neuroinflammation/microglia cells	<30 µm	Intermediate, similar to RNFL	No	Throughout all retinal layers

## Abbreviations

The following abbreviations are used in this manuscript:

AMD	Age-related macular degeneration
DME	Diabetic macular edema
DR	Diabetic retinopathy
ELM	External limiting membrane
GA	Geographic atrophy
HRD	Hyperreflective dots
HRF	Hyperreflective retinal foci
HRS	Hyperreflective retinal spots
OCT	Optical coherence tomography
ONL	Outer nuclear layer
OPL	Outer plexiform layer
PED	Pigment epithelium detachment
RPE	Retinal pigment epithelium
SRD	Serous retinal detachment
VEGF	Vascular endothelium growth factor
WHO	World Health Organization
